# “Efforts to Reprioritise the Agenda” in China: British American Tobacco's Efforts to Influence Public Policy on Secondhand Smoke in China

**DOI:** 10.1371/journal.pmed.0050251

**Published:** 2008-12-23

**Authors:** Monique E Muggli, Kelley Lee, Quan Gan, Jon O Ebbert, Richard D Hurt

**Affiliations:** 1 Mayo Clinic, Rochester, Minnesota, United States of America; 2 Centre on Global Change and Health, London School of Hygiene & Tropical Medicine, London, United Kingdom; 3 Center for Tobacco Control Research and Education, University of California San Francisco, San Francisco, California, United States of America; University of Queensland, Australia

## Abstract

**Background:**

Each year, 540 million Chinese are exposed to secondhand smoke (SHS), resulting in more than 100,000 deaths. Smoke-free policies have been demonstrated to decrease overall cigarette consumption, encourage smokers to quit, and protect the health of nonsmokers. However, restrictions on smoking in China remain limited and ineffective. Internal tobacco industry documents show that transnational tobacco companies (TTCs) have pursued a multifaceted strategy for undermining the adoption of restrictions on smoking in many countries.

**Methods and Findings:**

To understand company activities in China related to SHS, we analyzed British American Tobacco's (BAT's) internal corporate documents produced in response to litigation against the major cigarette manufacturers to understand company activities in China related to SHS. BAT has carried out an extensive strategy to undermine the health policy agenda on SHS in China by attempting to divert public attention from SHS issues towards liver disease prevention, pushing the so-called “resocialisation of smoking” accommodation principles, and providing “training” for industry, public officials, and the media based on BAT's corporate agenda that SHS is an insignificant contributor to the larger issue of air pollution.

**Conclusions:**

The public health community in China should be aware of the tactics previously used by TTCs, including efforts by the tobacco industry to co-opt prominent Chinese benevolent organizations, when seeking to enact stronger restrictions on smoking in public places.

## Abstract 2

Monique Muggli and colleagues study British American Tobacco (BAT) internal documents and find that from the mid 1990s BAT pursued a strategy aimed at influencing the public debate on secondhand smoke in China.

## Editors' Summary

**Background.:**

Each year, about one million people die in China from tobacco-caused diseases, including cancer, heart disease, and lung disease. Although most of these deaths occur among smokers—300 million people smoke in China, accounting for one-third of the global “consumption” of cigarettes—more than 100,000 deaths from tobacco-related causes occur annually among the 540 million Chinese people who are exposed to secondhand smoke. Tobacco smoke contains 4,000 known chemicals, 69 of which are known or probable carcinogens, and, when it is produced in enclosed spaces, both smokers and nonsmokers are exposed to its harmful effects. The only effective way to reduce tobacco smoke exposure indoors to acceptable levels is to implement 100% smoke-free environments—ventilation, filtration, and the provision of segregated areas for smokers and nonsmokers are insufficient. Importantly, as well as protecting nonsmokers from secondhand smoke, the implementation of smoke-free public places also reduces the number of cigarettes smoked among continuing smokers, increases the likelihood of smokers quitting, and reduces the chances of young people taking up smoking.

**Why Was This Study Done?:**

Article 8 of the World Health Organization's Framework Convention on Tobacco Control (FCTC; an international public-health treaty that seeks to reduce tobacco-caused death and disease) calls on countries party to the treaty to protect their citizens from secondhand smoke exposure. China became a party to the FCTC in 2005 but restrictions on smoking in public places in China remain limited and ineffective. Previous analyses of internal tobacco industry documents have revealed that transnational tobacco companies (TTCs) have used a multifaceted approach to undermine the adoption of restrictions on smoking in many countries. TTCs have been shown to influence media coverage of secondhand smoke issues and to promote ineffective ventilation and separate smoking and nonsmoking areas in restaurants, bars, and hotels (so-called “resocalization of smoking” accommodation principles) with the aim of undermining smoke-free legislation. In addition, TTCs have created organizations interested in non-tobacco-related diseases to draw attention away from the public-health implications of secondhand smoke. In this study, the researchers ask whether TTCs have used a similar approach to undermine the adoption of restrictions on smoking in China, one of the most coveted cigarette markets in the world by the major TTCs.

**What Did the Researchers Do and Find?:**

The researchers analyzed internal corporate documents produced by British American Tobacco (BAT; the predominant TTC in China) in response to litigation against major cigarette manufacturers stored in document depositories in Minnesota, USA and Guildford, UK. Among these documents, they found evidence that BAT had attempted to divert attention from secondhand smoke issues toward liver disease prevention by funding the Beijing Liver Foundation (BFL) from its inception in 1997 until at least 2002 (the most recent year that BAT's corporate records are available for public review). The researchers also found evidence that BAT had promoted “resocialization of smoking” accommodation principles as a “route to avoid smoking bans” and pushed ventilation and air filtration in airports and in establishments serving food and drink. Finally, the researchers found evidence that BAT had sought to “present the message that ‘tobacco smoke is just one of the sources of air polution [sic] and a very insignificant one compared with other pollutants'” through presentations given to the Chinese tobacco industry and media seminars aimed at Chinese journalists.

**What Do These Findings Mean?:**

These findings indicate that, beginning in the mid 1990s and continuing until at least 2002, BAT has followed an intensive, multi-pronged strategy designed to undermine the health policy agenda on secondhand smoke in China. Given their findings, the researchers suggest that BFL and other charitable organizations in China must be wary of accepting tobacco money and that measures must be taken to improve the transparency and accountability of these and other public organizations. To meet FCTC obligations under Article 5.3 (industry interference), policy makers in China, they suggest, must be made aware of how BAT and other TTCs have repeatedly sought to influence health policy in China by focusing attention toward the adoption of ineffective air filtration and ventilation systems in hospitality venues rather than the implementation of 100% smoke-free environments. Finally, Chinese policy makers and the media need to be better informed about BAT's long-standing attempts to communicate misleading messages to them about the health effects of secondhand smoke.

**Additional Information.:**

Please access these Web sites via the online version of this summary at http://dx.doi.org/10.1371/journal.pmed.0050251.

- The World Health Organization's Regional Office for the Western Pacific provides smoking statistics for China and other countries in the region
- The World Health Organization provides information on the health problems associated with secondhand smoke, about its Tobacco Free Initiative (available in several languages), and about the Framework Convention on Tobacco Control (also available in several languages)
- MedlinePlus provides links to information about the dangers of secondhand smoke (available in English and Spanish)
- The UK National Health Service Smokefree Web site provides information about the advantages of giving up smoking, how to give up smoking, and the dangers associated with secondhand smoke
- British American Tobacco documents stored in the Minnesota and Guildford Depositories, including those analyzed in this study, can be searched through the British American Tobacco Documents Archive

## Introduction

With one-third of the world's smokers, China has remained one of the most coveted cigarette markets in the world [1,2]. Approximately one million people die each year in China from tobacco-caused diseases [3]. By 2025, annual tobacco-caused deaths in China are expected to reach two million if smoking rates do not decline [4]. Given the high number of smokers in China, secondhand smoke (SHS) is a major public health problem. The prevalence of SHS exposure in nonsmokers was 53% in 1996 and 52% in 2002 [5]. In 2007, the Ministry of Health estimated that 540 million Chinese are exposed to SHS, resulting in more than 100,000 deaths annually [6].

The implementation of smoke-free public places has been shown to reduce the number of cigarettes smoked per day among continuing smokers, increase the likelihood that smokers will quit smoking, reduce the chances of a young person initiating smoking, and decrease health risks posed to nonsmokers [7–11]. The Chinese Ministry of Health estimates that only 35% of Chinese citizens know that SHS presents health risks to nonsmokers [6]. As of 2007, 154 out of 600 Chinese cities have passed smoke-free legislation regulating public places [12]. However, restrictions on smoking are rarely enforced [13,14]. Moreover, during the 2008 Olympic Games in Beijing, only selected indoor workplaces such as hotels, restaurants, taxis, and other tourist sites were made temporarily smoke free [15].

Previous analyses of internal tobacco industry documents have described the global strategy of transnational tobacco companies (TTCs) to undermine scientific evidence demonstrating the harmful health effects of SHS through covertly recruiting scientists in China [16–20] and globally [21–25], influencing media coverage of SHS issues, and creating front organizations [21,23,26–29]. Previous research has also shown that TTCs have alternatively promoted ineffective ventilation and separate seating for smokers and nonsmokers in hospitality venues with the aim of undermining smoke-free legislation [30,31].

In 2005, China became a party to the WHO Framework Convention on Tobacco Control (FCTC), the world's first international public health treaty, which seeks to reduce tobacco-caused death and disease [32]. Article 8 of the FCTC specifically provides that parties to the treaty protect its citizens from SHS exposure and recognize that scientific evidence unequivocally establishes that SHS causes disease and death [33]. As China moves to fulfil its obligations under the FCTC, an understanding of the tactics previously used by BAT to prevent effective restrictions on smoking is warranted.

This paper examines the efforts of British American Tobacco (BAT), the predominant TTC in China [1], to undermine the health policy agenda in China on SHS. Documents suggest that BAT pursued a multi-pronged strategy to divert government and media attention away from SHS issues and toward liver disease prevention initiatives through the funding and promotion of the China-based Beijing Liver Foundation (BLF). In addition to BAT's efforts through the BLF, documents show that the company sought to introduce air filtration and ventilation technology and to promote so-called “resocialisation of smoking” principles. These principles were used to circumvent smoke-free legislation and train Chinese media and representatives of the state tobacco monopoly industry on its corporate messages that SHS is an insignificant contributor to the larger air pollution problem. An understanding of the tactics employed by BAT demonstrates the need for continued vigilance by the public health community and policy makers in China seeking to strengthen smoke-free legislation.

### Background

Previous research has described diversionary tactics by BAT and other TTCs aimed at undermining the 8th World Conference on Tobacco or Health (WCTOH) held in Buenos Aires in 1992. TTCs sought to distract media attention away from the 8th WCTOH and toward staged events such as music concerts and soccer matches promoting childhood immunization and HIV/AIDS awareness [34,35]. Previous research has also detailed TTCs' efforts to create the “ETS [Environmental Tobacco Smoke] Consultants Program” in the 1980s and mid 1990s, through which scientists were trained and managed by tobacco industry lawyers to support the industry's position on SHS throughout Asia, Europe, South and Central America, and the United States [16,21–23]. Additionally, TTCs recruited and paid Asian scientists to publish in industry-funded journals proclaiming that SHS exposure constituted minimal health risks [16,23–25]. Within this context, this paper finds that, in China, BAT sought to shift attention away from tobacco-related health concerns to liver disease by funding and promoting the Beijing Liver Foundation (BLF).

## Methods

This paper analyzes internal corporate documents produced in response to litigation involving the major tobacco manufacturers. The history and public availability of these records have been described previously [36–41]. Online searches of documents produced by BAT were conducted at the British American Tobacco Document Archive Web site (http://bat.library.ucsf.edu). Documents were also searched on-site at the Guildford Depository in the United Kingdom over multiple visits from 2000 to 2006.

On-line searches followed an iterative model, initially combining terms such as “China”, “Beijing”, “health promotion”, “hepatitis”, and “liver disease” with Boolean operators. This was followed by more specific searches using names of BAT projects and personnel. Additionally, files belonging to individuals directly communicating with BAT's subsidiary, BAT China, were searched. All relevant documents were analyzed to create a historical and thematic narrative. Industry publications, newspapers, academic journals, policy documents, and legislation were used to contextualize and triangulate findings.

The limitations of using internal tobacco company records in research have been described previously [34,41]. Limitations most relevant to this research include the following: (1) Access to documents originating from BAT's Chinese subsidiary is limited because BAT China was not named as a defendant in the lawsuits in which these documents were produced; (2) it was difficult to triangulate BAT's corporate records with public sources due to limited access to information within China; and (3) BAT's documents produced in litigation are currently limited to those records created before 2002.

## Results

### Diverting Attention from SHS Issues toward Liver Disease Prevention

BAT China funded the BLF (renamed the Beijing Health Promotion Society in 1999) from its inception in 1997 to at least 2002 [42] (2002 is the most recent year that BAT's corporate records are currently available for public review). The BLF was founded in 1997 [43] by the Soong Ching Ling Foundation (SCLF) [44], whose stated mission is to further the well-being of Chinese children through various education programs [44]. BAT characterized the organization as one of “the antis [anti-smoking group] in the PRC” [45]. Documents disclose that BAT initially funded BLF, in part, to distract attention away from the 10th WCTOH held in Beijing in 1997 [46]: “Whilst smoking & health issues are of concern to the PRC there are other issues which we believe should be of greater significance to the PRC and the WHO including hepatitis which is very prevalent in China and a major health concern. We are therefore supporting the Liver Foundation to raise the profile of this important issue” [47].

Further, BAT sought to use the BLF to influence the priorities of the SCLF and Ministry of Public Heath (MPH). According to a 1999 report entitled *Beijing Liver Foundation Report*, BAT stated: “The [Beijing Liver] Foundation was formed with the support of British American Tobacco China in an effort to reprioritise the agenda of the Ministry of Public Health (MPH) and Soong Ching Ling Foundation, the two most active antis, to tackle the No. 1 infectious disease in China and to divert the public attention from smoking and health issues to liver diseases” [42].

Through BLF, BAT sought to “lobby the Ministry of [Public] Health to maintain a perspective on health issues; hepatitis is the number one killer disease in China” [45]. BAT sponsored BLF as a means of communicating with the MPH, because “BAT cannot, credibly, directly communicate with the Ministry. We sponsor the Liver Foundation programs so that the Ministry can, hopefully, re-prioritize health issues” [18].

To further foster a relationship with the MPH through BLF, several Chinese regulators were invited as consultants to the BLF, including the vice minister of the MPH and the deputy chairman of SCLF [42]. Moreover, by cosponsoring youth smoking prevention programs with SCLF [48], BAT hoped to gain endorsement of such programs and strengthen government relations [45,49]. The sincerity of these overtures, however, warrant doubt given the youth-targeted marketing activities in specific brand plans during this time such as its “sponsorship of Kent Pop Music Chart to circumvent market restrictions” and targeting of “new Generation smokers” in restaurants and bars [50].

During 1997–2002, BLF undertook several hepatitis-focused health promotion and disease prevention initiatives throughout China [51]. Although many of the programs were seemingly designed to benefit public health, BAT's influence over BLF's program is readily apparent. Documents disclose that, while many of BLF's activities appear to be legitimate liver disease prevention programs, others promoted the company's agenda of circumventing smoke-free initiatives.

### Beijing Liver Foundation's Liver Disease Prevention and Health Promotion Programs

BLF launched several educational programs using different approaches to generate public awareness of the causes, symptoms, diagnosis, and treatment of liver diseases [52]. These initiatives included public talks on self-care; TV and radio programs concentrating on the epidemiology, prevention, and treatment of chronic hepatitis; hotline and internet services providing medical consultation; major liver disease exhibitions throughout China; and calligraphy and drawing competition for children in schools [52]. For example, one news report detailed BLF's exhibition program in 1997: “An educational exhibition about the disease was held in late August by the Beijing Liver Foundation, a non-governmental organization under the Soong Ching Ling Foundation. Children were invited to the Museum of Chinese Revolution free of charge, where vivid pictures and video programmes presented a living lecture on the disease. The three-day exhibition was only part of the programme organized by the liver foundation. A five-part TV serial promoting knowledge about liver diseases was produced by the foundation in May” [53].

In 2000, another large exhibition on hepatitis prevention and treatment, held in Kunming, in Yunnan province, was used by BAT China as a publicity opportunity: “Senior officials from the provincial and municipal governments, local legislators, leading members of the provincial healthcare administration, representatives from the Madam Song Foundation, the Disease Control Division of the Ministry of Health, the Beijing Health Promotion Council and BAT China attended the ribbon-cutting ceremony. Ms. Li Xiaofen, Representative of BAT China, presented 50,000 cartoon books on the elementary knowledge about hepatitis to the provincial health administration on behalf of the organizing committee of the show” [54].

### Beijing Liver Foundations Programs Promoting BAT's Position on SHS in China

Documents show that BAT successfully used BLF to push its agenda on SHS issues in China by promoting SHS studies funded by BAT on BLF's Web site [55,56]. In 1999, BLF introduced a “journal of health” [57] on its Web site (www.wellness.com.cn), designed for medical consultation for liver diseases [55]. Also included on the Web site was “[P]ositive ETS studies… [BAT] positions on smoking and health issues, and balanced views on lung cancer diseases” [55]. BAT also noted that BLF's Web site “[P]rovided us [BAT] a channel to reach our customers” [56]. A 1998 internal memo from BAT China indicated that the company would “continue to make use of this web[site] to post more ETS studies” [58]. BAT reported an average of 20,000 visits per day to this Web site since its establishment in June 1998 [58]. In 2000, the following statement was to be released on the Web site: “No direct correlation has been found between lung cancer risks and exposure to environmental tobacco smoke” [59].

The Web site appears to be no longer active on the internet at the time of this writing.

By 2001, in addition to Web-based activities, BAT directly funded at least two SHS research projects through BLF. First, a project entitled “Hazards to non-smokers exposed to ETS,” employed a panel of experts from the China Preventive Medicine Academy to translate and analyze the contents of 735 “articles” and “professional magazines” from 1994–1998 related to SHS, such as: “[T]he density analysis of indoor ETS content; physical, chemical and physiological analyses of ETS; epidemiological data on the impact of ETS on the health of different age groups; and evaluation of lung cancer risks to non-smokers exposed to ETS” [60].

Their findings were presented at an “Indoor Air Quality Forum” in Beijing and reported by “major media including CETV” [61]. The panel concluded the following: “[U]nder normal conditions, passive smoking is less risky than active smoking; both the relative risk and the attributable risk of lung cancer from passive smoking are small; due to reasons such as poor exposure characterization, lack of control for certain confounding factors, and the fact that biomarkers can only reflect recent exposure to ETS, study results vary considerably in risks of passive smoking and there has been no international consensus on this issue so far” [62].

The second project, entitled “Study on factors related with lung cancer risks to non-smoker women in China,” sought to conduct a case-control study of 3,000 nonsmoking Chinese women with the aim to “[H]elp the public [become] better acquainted with the genuine elements leading to increased female lung cancers [sic] cases and enhance their awareness of the danger that environmental pollutants may cause to residents, women residents in particular” [60].

Although several meetings between officials and medical experts from the SCLF, China Preventive Medicine Academy, and BLF had taken place after 1999, the report noted that because of restructuring of the Chinese health care system the project leaders delayed implementation and the project was expected to finish at the end of 2002 [63]. We were unable to verify whether this project was completed.

### “Resocialisation of Smoking” [45]: Accommodation Principles Aimed at Circumventing Smoke-free Legislation

Documents reviewed also describe additional strategies aimed at weakening secondhand smoke policies in China. Similar to previous efforts in the UK [31] and other markets [30,64], BAT sought to “promote air filtration and make it normal practice” [65] by using “accommodation” efforts, which refer to the TTC strategy of lobbying for separate seating for smokers and nonsmokers and promoting ventilation and air filtration technology for hospitality venues [30,64]. Also, BAT has pushed accommodation efforts as a “route to avoid smoking bans” [66]. Accordingly, BAT's 2000–2002 “resocialisation” initiatives contained in its Asia Pacific North Company Plan called for: “‘Resocialisation of Smoking' efforts …aimed to promote the concept of accommodation to lobby for the delay of further restrictions on public smoking” [45].

BAT's plan for “resocialising” smoking in China included pushing ventilation and air filtration in airports and hotel, restaurant, and casino (“HORECA”) outlets [45]. BAT then sought to train Chinese hospitality groups in an effort to “support…lobbying efforts for self regulation as an alternative to restrictive government regulations” [67]. In May 2000, Adrian Payne (BAT International Scientific Affairs Manager) reported that the company was successful in creating ventilated airport smoking lounges at several airports in Asia including one in Shanghai [68].

The air filtration systems installed in China were manufactured by Colt International and were used by BAT to serve the dual purpose of circumventing restrictions on smoking and allowing the company direct communications with customers through branding opportunities in a restrictive advertising environment [69,70]. Previous research reported that, during the mid 1990s, BAT installed Colt filtration systems worldwide despite its recognizing the Colt filters as being ineffective at removing harmful constituents from the air [31].

### BAT's “Knowledge Transfer” [71] to the Chinese Tobacco Industry and Chinese Media on SHS Messages

Through presentations in the mid 1990s to the Chinese tobacco industry and media seminars aimed at Chinese journalists, BAT also sought to “present the message that ‘tobacco smoke is just one of the sources of air polution [sic] and a very insignificant one compared with other pollutants'” [72]. However, documents indicate that company efforts to communicate directly with the public about SHS were hindered by perceived constraints on free speech in China [73]. Thus, BAT began training its BAT China employees in 1996 [74] on developing media programs that would position SHS as “just one of the sources of air pollution and at a very slight degree compared with other pollutants” [75]. Also, “At the time when Chinese government and the publics [sic] attach much attention to environment protection while lacking enough funds, it is the heyday to take part in the publicity programme organized by environment administrations and to raise funds to import advanced technology on pollution control, especially on air pollution. And the publics [sic] are expected to move their eyes from ETS to the problem of air pollution” [75].

According to BAT China management, employees “did not know any more about these issues than from what they read in the newspapers” [76]. BAT China employees were briefed by scientific managers and lawyers from BAT headquarters regarding the company's positions on indoor air quality [74]. During this SHS “issues training,” employees were primarily exposed to research and public statements made by scientists who were paid by the tobacco industry [77,78], including some of the same scientists and journalists who were previously recruited globally by the TTCs to “keep the ETS controversy alive” [16,22–24,29].

BAT also sought to train representatives of the Chinese State Tobacco Monopoly Administration (STMA) given its control of 98% of the Chinese market. BAT and BAT China engaged in a systematic “knowledge transfer” to the STMA as a way to communicate to media and policy makers that it could not directly reach [71,79]: “Briefings to STMA will be important since they are the most appropriate conduit to spread the message to a much wider audience, namely the media and through the media the general public and other government regulators. The plan is to continue to involve STMA in all communications initiatives as follows…. [a] seminar will be organized for STMA personnel on IAQ [indoor air quality], smoking and health issues in June [1997]…” [80].

By 2000, at least six “smoking and health seminars” directed at STMA were held by BAT [81]. BAT's first STMA briefing occurred in June 1997 in Beijing. BAT presented its view that: (1) there are insufficient data to conclude that SHS exposure is a risk factor for or cause of lung cancer [82] and heart disease [83]; (2) SHS exposure is not a cause of disease in children [84]; and (3) government regulations are not needed to protect public health [85]. BAT also presented the idea that SHS exposure is “not a health issue but a social issue” [86].

BAT concluded after its May 2000 seminar that its efforts aided in building a stronger relationship with the Chinese tobacco industry and regulators: “The seminar has strengthened communications between BAT and the Chinese tobacco industry. It has also strengthened communications between the tobacco industry and regulators. The seminar has helped the Chinese tobacco industry and regulators to better understand the latest development and activities of major antis in the world, which are of key importance for them to work out countering strategies” [87].

In addition to “briefing” the Chinese tobacco industry, BAT sought to influence the media through a number of seminars. In September 1996, BAT held a media seminar entitled “Indoor Air Quality and Ventilation Technology” in Urumqi as “a litmus test of [the] media's response to the subject matter, of obtaining balanced coverage and to contain the impact” of the 10th WCTOH [88]. The seminar began with an overview by BAT's then Director of Smoking Issues Chris Proctor who, contrary to a then decade of conclusive research showing that SHS causes disease and death, characterized SHS-related health consequences as an erratic concept subject to the whims of obscure media reports [89]: “Indoor air quality has recently become a very important topic throughout the Asia region. Virtually every day a story appears alleging a new threat to human health which is present in our home or offices. One day the story is about emissions from furniture; the next day the story is about a group of workers afflicted with the mysterious ‘sick building syndrome'; the following day there will be a story about a disease supposedly associated with breathing the smoke from other people's cigarettes...Experts in air quality, with experience in both the Asia region and Western countries, were brought together to review the scientific evidence, to identify the real indoor air quality problems facing the Asia region, and to suggest solutions which are appropriate and practical” [90]. (Emphasis added.)

Media coverage in China at this time suggests that BAT had some success at framing SHS in terms of poor indoor air quality. For example, an article published in *Renmin Ribao (China Daily)* in December 1998 entitled “Study finds serious indoor air problem” stated that tests carried out by the government, BAT, and Healthy Buildings International showed that the “the main causes for typical indoor air problems in these buildings were inadequate ventilation and filtration, and insufficient maintenance of air-purification systems” and that “smoking was reported not an important factor influencing air quality” [91]. The article cited Zhang Kunmin, Secretary-General of the China Council for International Co-operation on Environment and Development, who is said to have stated that “the related research results would aid in policy-making” [91].

BAT continued to organize media SHS seminars to generate “positive and balanced coverage in major media” and to “post BAT views on the STMA website” until at least 2002 [45]. Documents show that BAT framed SHS within an indoor air pollution context so that it could directly advocate for air filtration and ventilation solutions. In conducting these media seminars, BAT did not disclose the past long-term consulting arrangement with “experts” who addressed the media.

## Discussion

This review of internal documents finds that, beginning in the mid-1990s, BAT pursued a multi-faceted strategy aimed at influencing and undermining the public debate on SHS issues in China. BAT sought to shift policy attention from SHS issues to liver disease prevention through the funding and promotion of the Beijing Liver Foundation. Several aspects of BAT's efforts to create and fund the BLF are notable. First, heart disease, cancer, and cerebrovascular disease account for two-thirds of deaths in China in the population ≥ 40 y of age, for which tobacco is the leading preventable risk factor [92]. In fact, these authors estimate that cigarette smoking is responsible for 7.9% of overall mortality in China, which is second only to hypertension at 11.7% [92]. Hepatitis B is endemic in China, but chronic liver disease and cirrhosis account for only 1.5% of total deaths, while chronic obstructive pulmonary disease alone accounts for 1.8% of total deaths [92]. Similarly, lung cancer alone accounts for more deaths in China than does primary liver cancer, a disease known to be caused by chronic hepatitis B infection [92]. As a distraction, then, BAT chose to focus on a disease that contributes a significant [93–95], but proportionally smaller disease burden and, most importantly, has little direct link to tobacco use. Moreover, internal documents describe the lobbying effort to blatantly misinform the MPH with the message that hepatitis was “the number one killer disease in China”[45]. Second, BAT had previously sought to distract public attention away from tobacco-caused disease in Latin America by promoting HIV/AIDS awareness and childhood immunizations to counter attention to a single event—the 8th WCTOH in Argentina. In China, BAT's strategy was initiated for the 10th WCTOH, but it also used the BLF as a vehicle for influencing SHS issues over subsequent years. Third, while previous analyses have shown that BAT and other TTCs have worked extensively through front groups to influence scientific and policy debates, notably where TTCs have lacked credibility [34,96,97], in China, documents disclosed in litigation show that BAT was successful at using BLF to infiltrate programs conducted by a respected charitable organization targeted by the company as among “the antis”.

This analysis of internal documents also shows that BAT promoted air filtration and ventilation technologies as part of a “resocialisation of smoking” effort in anticipation of potential restrictions on smoking. BAT achieved this by engaging in a “knowledge transfer” of the industry-based message to the Chinese tobacco industry and media that SHS is an insignificant contributor to the larger issue of air pollution. In doing so, BAT advocated the position that SHS exposure in hospitality venues did not warrant regulation. Instead, because SHS was, according to BAT, an insignificant source of pollution, the issue could be addressed by so-called “accommodation” efforts.

These findings raise several policy implications. Because BAT was able to incorporate its messages on SHS into the BLF's programs targeted at liver disease prevention, charitable organizations in China must be wary of accepting tobacco industry funding. While funding may appear to be for scientific or benevolent purposes and seemingly unrelated to tobacco use, documents reviewed here reveal that industry motives can be well hidden. Measures to improve transparency and accountability of public organizations, notably their association with the tobacco industry, should continually be improved worldwide. Disclosure of this kind will lessen the capacity of the tobacco industry to covertly use local institutions to pursue its interests. In doing so, parties to the FCTC may act to fulfil their obligations under Article 5.3 of the FCTC to protect implemented public health policies from industry influence [98].

Additionally, as policy makers consider the adoption of smoke-free public places, such as the Beijing smoke-free initiative [99], Chinese public health advocates should be aware of how BAT and other TTCs have repeatedly sought to focus attention toward the adoption of ineffective air filtration and ventilation systems in hospitality venues. Already, one manufacturer of “Clean Air Smoking Area” technology has expanded its distribution to the Chinese market [100,101]. Policy makers in China should be encouraged to follow WHO recommendations and guidelines concerning Article 8 of the FCTC on the implementation of 100% smoke-free environments rather than the introduction of ineffective ventilation technologies [102,103].

Finally, Chinese policy makers and the media need to be better informed of BAT's decade-long initiative to communicate misleading messages on the health effects of SHS. Public statements in recent years by the Chinese tobacco industry suggest that these messages are being disseminated in China. In 2006, despite over two decades of international scientific consensus on the harmful health effects of SHS [89], the STMA stated that more research was needed to determine the consequences of SHS exposure: “Exposure to tobacco smoke can cause different consequences depending on amount of tobacco smoke exposed to, and individual's physical fitness. Only through thorough more detailed and targeted researches [sic] can we come to scientific and fair results” [104].

Public health advocates in China should expose such misinformation as part of their efforts to address the health effects of SHS.

## Abbreviations

BAT: British American Tobacco
BLF: Beijing Liver Foundation
ETS: environmental tobacco smoke
HORECA: Hotel, Restaurants, and Casinos
IAQ: indoor air quality
MPH: Ministry of Public Health
SCLF: Soong Ching Ling Foundation
SHS: secondhand smoke
STMA: State Tobacco Monopoly Administration
TTC: transnational tobacco company
WCTOH: World Conference on Tobacco or Health
